# Inhibition of Autophagy Enhances the Antitumor Effect of Thioridazine in Acute Lymphoblastic Leukemia Cells

**DOI:** 10.3390/life11040365

**Published:** 2021-04-20

**Authors:** Carina Colturato-Kido, Rayssa M. Lopes, Hyllana C. D. Medeiros, Claudia A. Costa, Laura F. L. Prado-Souza, Letícia S. Ferraz, Tiago Rodrigues

**Affiliations:** 1 Centro de Ciências Naturais e Humanas (CCNH), Universidade Federal do ABC (UFABC), Santo André 09210-580, São Paulo, Brazil; carina.colturato@ufabc.edu.br (C.C.-K.); lopes.rayssa@ufabc.edu.br (R.M.L.); hyllana.medeiros@ufabc.edu.br (H.C.D.M.); laura_prado2013@hotmail.com (L.F.L.P.-S.); letisbloom@hotmail.com (L.S.F.); 2 Centro Interdisciplinar de Investigação Bioquímica (CIIB), Universidade de Mogi das Cruzes (UMC), Mogi das Cruzes 08780-911, São Paulo, Brazil; claudia.bio@outlook.com

**Keywords:** acute lymphoblastic leukemia, apoptosis, autophagy, viability, thioridazine

## Abstract

Acute lymphoblastic leukemia (ALL) is an aggressive malignant disorder of lymphoid progenitor cells that affects children and adults. Despite the high cure rates, drug resistance still remains a significant clinical problem, which stimulates the development of new therapeutic strategies and drugs to improve the disease outcome. Antipsychotic phenothiazines have emerged as potential candidates to be repositioned as antitumor drugs. It was previously shown that the anti-histaminic phenothiazine derivative promethazine induced autophagy-associated cell death in chronic myeloid leukemia cells, although autophagy can act as a “double-edged sword” contributing to cell survival or cell death. Here we evaluated the role of autophagy in thioridazine (TR)-induced cell death in the human ALL model. TR induced apoptosis in ALL Jurkat cells and it was not cytotoxic to normal peripheral mononuclear blood cells. TR promoted the activation of caspase-8 and -3, which was associated with increased NOXA/MCL-1 ratio and autophagy triggering. AMPK/PI3K/AKT/mTOR and MAPK/ERK pathways are involved in TR-induced cell death. The inhibition of the autophagic process enhanced the cytotoxicity of TR in Jurkat cells, highlighting autophagy as a targetable process for drug development purposes in ALL.

## 1. Introduction

Acute lymphoblastic leukemia (ALL) is a malignant disorder of lymphoid progenitor cells that affects both children and adults [1]. T cell acute lymphoblastic leukemia (T-ALL) is one of the most aggressive hematologic malignancies, which is reported in 10–15% of pediatric and 25% of adult ALL cases [2]. Despite the high cure rate, drug resistance still remains as a significant clinical problem, which stimulates the development of novel therapeutic strategies and drugs to improve the outcome of this disease.

The phenothiazine derivative thioridazine (TR) is an antipsychotic drug that has been used in medicine in the treatment of psychosis and schizophrenia [3]. Several recent studies showed the cytotoxicity of TR in several types of cancer cells in vitro [4,5,6]. A structure-activity study in hepatocarcinoma cells revealed that TR, a piperidine derivative, was the most potent phenothiazine to induce cell death [6]. A recent review summarized the anticancer activity of fluphenazine, perphenazine, and prochlorperazine [7]. Previous studies suggested that the inhibition of the mitochondrial DNA polymerase, impairment of ATP production, and PI3K/AKT/mTOR signaling pathway are among the mechanisms contributing to TR-induced cell death [8,9,10]. However, there are few mechanistic studies about the cytotoxicity of phenothiazines in leukemia. Chlorpromazine suppressed the growth of primary AML cells and AML cells with mutated tyrosine kinase receptor [11]. Yet, in AML cells, TR induced cell death promoting cytoskeletal remodeling of blast cells selectively in variant t (6;11) AML cells involving Ca^2+^ overload, ROS production, and mitochondrial dysfunction [12]. Although several mechanisms have been proposed to explain its cytotoxicity, the molecular mechanisms underlying TR-induced apoptosis has not been completely understood, especially in acute lymphoblastic leukemia.

The clinical use of TR as a neuroleptic is associated with the development of relevant side effects in some patients, such as dysrhythmia and sudden death [13]. Thus, the combination of lower TR concentrations with other anticancer drugs in a combined chemotherapy might reduce the incidence of undesirable side effects and improve the possible anticancer effect. In this regard, the modulation of autophagy has been proposed as a promising therapeutic strategy in cancer therapeutics [14,15]. Autophagy is an evolutionarily conserved pathway that mediates the degradation of cellular components, such as proteins and organelles, and contributes to cellular homeostasis [16]. However, it was shown that tumor cells present an increased autophagic capacity and flux compared to normal cells [17]. Several drugs employed in cancer therapy induce a protective autophagy response, which can contribute to therapeutic resistance [18], pointing to the pharmacologic inhibition of autophagy as a promising approach to promote/increase tumor cell death elicited by other drugs [19].

In this study, the cytotoxicity of TR against a human T-ALL model was evaluated in vitro and the role of autophagy in the observed cell death. Our data showed that TR induces a potent and selective cell death in T-ALL cells in vitro and also that autophagy inhibition potentiates its cytotoxicity, constituting a promising strategy for ALL chemotherapy.

## 2. Materials and Methods

### 2.1. Reagents

**Figure 1:**
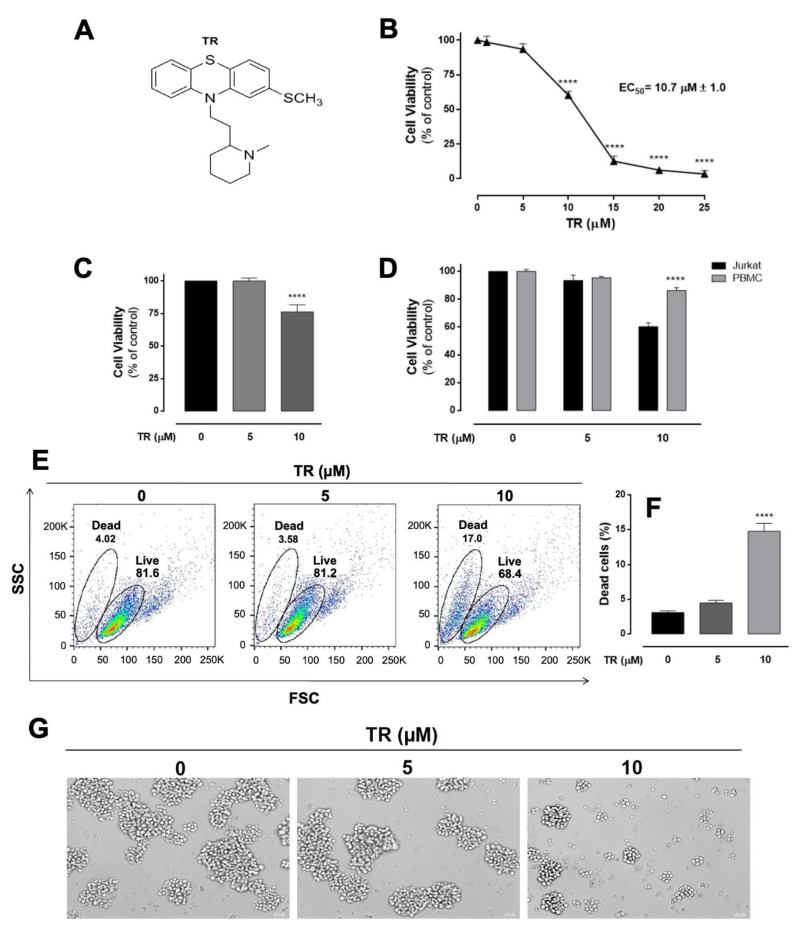
Enhancement of the cytotoxicity of TR and selectivity against human leukemia Jurkat cells compared to human normal blood cells. (**A**) Molecular structure of the thioridazine. (**B**) Cell viability was assessed by the MTT reduction test. Cells were incubated with increasing TR concentrations (0–25 µM) for 24 h. The EC_50_ value for TR was calculated as 10.7 μM. (**C**) Cell viability was also assessed by the trypan blue exclusion assay. **** (*p* < 0.0001) indicates a difference from control (absence of the drug). (**D**) Cell viability assessed by MTT reduction test in normal and leukemia cells obtained with 5 and 10 µM TR. The human peripheral blood mononuclear cells (PBMC) were stimulated with 5 µg/mL phytohemagglutinin. The percentage of viable cells was calculated in relation to control (untreated), considered as 100%. The results are presented as the mean ± SEM of at least three independent experiments performed in triplicate. **** Statistically different from Jurkat cells (*p* < 0.0001). (**E**) Changes in cell size and granularity (FSC × SSC parameters). Representative dot plots of at least three independent experiments performed in duplicate. (**F**) Quantification of dead cells based on FSC and SSC parameters. The results are presented as the mean ± SEM of at least three independent experiments performed in duplicate. **** (*p* < 0.0001) indicates a difference from control (absence of the drug). (**G**) Morphological alterations of Jurkat cells assessed by optical microscopy (400× magnification, scale bar 20 µM).

Thioridazine (10-[2-(1-methyl-2-piperidyl) ethyl]-2-methyl-thiophenothiazine) (Figure 1A), 3-methyladenine, leupeptin, bafilomycin A1 and chloroquine were purchased from Sigma-Aldrich, St. Louis, MO, USA. Rapamycin was obtained from Selleck Chemicals, Houston, TX, USA. LY294002 was obtained from Cell Signaling Technology, Danvers, MA, USA. Hoechst 33,258 and LysoTracker Green were obtained from Thermo Fisher Scientific, Waltham, MA, USA. Antibodies against AKT (pan) (#4691), Ambra-1 (#24907), AMPKα (#95832), Atg5 (#12994), Atg7 (#8558), BAK (#3814), BAX (#5023), BCL-xL (#2764), BCL-2 (#15071), BIM (#2933), B-Raf (#14814), caspase-3 (#9665), cleaved caspase-3 (Asp175) (#9661), cleaved caspase-8 (#9496) (Asp391), Lamp2 (#49067), LC3A/B (#12741), mTOR (#2983), NOXA (#14766), p-AKT (Ser473) (#4060), p-AMPKα (Thr172) (#2535), p-Beclin-1 (Ser15) (#13825), p-B-Raf (Ser445) (#2696), p-ERK1/2 (Thr202/Tyr204) (#9101), p-MEK1/2 (Ser217/221) (#9154), p-mTOR (Ser2448) (#2971), p-PI3K (Tyr199/458) (#4228), Ras (#3965), SQSTM1/p62 (#8025), ULK (#8054), β-actin (#3700), anti-mouse IgG HRP-linked (#7076), and anti-rabbit IgG HRP-linked (#7074) were obtained from Cell Signaling Technology, Danvers, MA, USA. Antibodies against Beclin-1 (612112), BID (611528), MCL-1 (559027) and PI3K (610045) were from BD Biosciences, San Jose, CA, USA.

### 2.2. Cell Culture

The Jurkat cell line (human acute lymphoblastic leukemia) was provided by Prof. Edgar J. Paredes-Gamero (UFMS, Brazil) and tested to be mycoplasma-free by indirect staining with Hoechst 33258. Cells were grown in RPMI-1640 medium (Sigma-Aldrich, St. Louis, MO, USA) pH 7.4, supplemented with 10% fetal bovine serum (Gibco, Invitrogen, Carlsbad, CA, USA), 100 U/mL penicillin, and 100 μg/mL streptomycin, in a 5% CO_2_ atmosphere at 37 °C (Panasonic MCO-19AIC, Kadoma, Osaka, Japan). For the experiments, cells were centrifuged (160× *g* for 10 min) and suspended in supplemented RPMI medium.

### 2.3. Cell Viability Assays

The cytotoxicity of the thioridazine was screened first by using the MTT reduction test and confirmed by the trypan blue exclusion assay in human leukemia Jurkat cells and normal peripheral blood mononuclear cells (PMBC), as previously described [6]. When used, 3-methyladenine, LY294002, leupeptin, rapamycin, bafilomycin A1, and chloroquine were preincubated 1 h before adding TR. Cell viability was calculated in relation to the control (absence of the drug), considered as 100%. The half maximal effective concentration (EC_50_) was calculated as described elsewhere [6].

### 2.4. Annexin V-FITC/PI Staining

Cells (1.0 × 10^5^/mL) were seeded in 24-well microplates in the presence of 10 µM TR for 24 h. Then, the cells were centrifuged (160× *g* for 10 min) and suspended in 50 μL of binding buffer (0.14 M NaCl, 2.5 mM CaCl_2_, 0.01 M HEPES, pH 7.4). Apoptosis was assessed by using the FITC Annexin V Apoptosis Detection Kit I (#556547, BD Biosciences, San Jose, CA, USA) according to the manufacturer’s instructions. The fluorescence emission was acquired a FACS Canto II flow cytometer (BD Biosciences, San Jose, CA, USA), acquiring 10,000 events per sample using a Coherent Sapphire 488-20 solid state blue laser with excitation at 488 nm, dichroic mirror 502 LP, bandpass filter 530/30 for the FITC fluorescence channel and dichroic mirror 556 LP, bandpass filter 585/42 for the PI fluorescence channel. Data analysis and graphs were completed using FlowJo vX.0.7 software (Ashland, OR, USA).

### 2.5. Mitochondrial Transmembrane Potential (ΔΨ)

Cells (1.0 × 10^5^/mL) were plated on 24-well microplates in the presence of 10 µM TR for 24 h. Then, cells were centrifuged (160× *g* for 10 min) and suspended in 1.0 mL of PBS. Cells were incubated with 100 nM tetramethylrhodamine methyl ester (TMRM) (Life Technologies, Invitrogen, Waltham, MA, USA) for 30 min in a 5% CO_2_ atmosphere at 37 °C. After, cells were washed in PBS and suspended in 0.3 mL of PBS. As a positive control, 20 μM carbonyl cyanide 3-chlorophenylhydrazone (CCCP) was used. Fluorescence emission was acquired with a FACSCanto II flow cytometer (BD Biosciences, San Jose, CA, USA), acquiring 10,000 events per sample using a Coherent Sapphire 488-20 solid state blue laser with an excitation at 488 nm, dichroic mirror 502 LP, bandpass filter 585/42 for the TMRM fluorescence channel. Data analysis and graphs were completed using FlowJo vX.0.7 software (Ashland, OR, USA).

### 2.6. Lysosomal Staining

Cells (1.0 × 10^5^/mL) were plated on 24-well microplates in the presence of 10 µM TR for 24 h. After incubation, cells were stained simultaneously with 5 µM Hoechst 33,342 and 50 nM LysoTracker Green in culture media for 30 min at room temperature. Fluorescence emissions were acquired in a widefield fluorescence microscopy system Leica AF6000 (Leica Microsystems, Wetzlar, Germany) using the set of cube filters A4 (Ex, 360/40; dichroic mirror, 400 nm; filter BP, 470/40) and L5 (Ex, 480/40; dichroic mirror, 505 nm; BP filter, 527/30), objective lens HCX APO U-V-I 100×/1.3 OIL, and camera DFC365FX.

### 2.7. Western Blot Analysis

After incubation with 10 µM TR for 12 and 24 h, cells were collected and lysed with RIPA buffer 1X (Thermo Fischer Scientific, Waltham, MA, USA) with a 1X protease/phosphatase inhibitor cocktail (Cell Signaling Technology, Danvers, MA, USA) and 1.0 mM PMSF (Cell Signaling Technology, Inc. USA). The protein concentration was determined by Lowry’s method, with BSA as standard to construct the analytical curve. Samples were heated at 95 °C for 5 min and stored at 4 °C. Aliquots (50–120 μg of protein) were loaded in a 12% sodium dodecyl sulfate-polyacrylamide gel and submitted to electrophoresis (SDS-PAGE). Separated proteins were transferred to nitrocellulose membranes (Bio-Rad Laboratories, Hercules, CA, USA) using the Trans-Blot Turbo Transfer system (Bio-Rad Laboratories, Hercules CA, USA). The nonspecific protein binding was avoided by incubating the membrane in 5% non-fat dry milk dissolved in TBST buffer for 1 h at room temperature. After, membranes were incubated overnight with the respective primary antibody (1:1000) in 1% BSA diluted in TBST at 4 °C. The membranes were then incubated with a specific secondary antibody conjugated with horseradish peroxidase (HRP), which was diluted 1:10,000 in 1% BSA for 1 h at room temperature (anti-mouse IgG HRP-linked or anti-rabbit IgG HRP-linked). Labeled proteins were detected using the chemiluminescent detection kit Pierce^™^ ECL Plus Substrate (Thermo Fischer Scientific, Waltham, MA, USA) and acquired with ChemiDoc^™^ MP Imaging system v5.0 (Bio-Rad Laboratories, Hercules, CA, USA). All Western blot images shown are representative of at least 3 independent experiments. In order to calculate the mean ± standard error of the mean (SEM), the optical density of the band was used as the unit of measure with Image Lab^™^ software, version 5.0 (Bio-Rad Laboratories, Hercules, CA, USA), and normalized by endogenous control *β*-actin. The quantification graphs considering all replicates, as well as, the representative crude Western blot membranes are presented in the Supplementary Material, Figures S1–S4.

### 2.8. Statistical Analyses

All data were expressed as the means ± SEM. Statistical analyses were performed using Prism 6.1 software (GraphPad Software Inc., San Diego, CA, USA). Data were tested using one-way ANOVA followed by the Tukey post hoc test with significance defined as * *p* < 0.05, ** *p* < 0.01, *** *p* < 0.001, and **** *p* < 0.0001.

## 3. Results

### 3.1. Thioridazine (TR) Exhibited Potent and Selective Cytotoxicity Against Human T-ALL Jurkat Cells

The cytotoxicity of TR was evaluated in vitro in cultured T-ALL Jurkat tumor cells and further compared with human peripheral mononuclear blood cells (PMBC) by the MTT assay after 24 h incubation. As observed in Figure 1B, TR significantly decreased the viability of Jurkat cells in a concentration-dependent manner, presenting the half-maximal effective concentration (EC_50_) value of 10.7 µM. Based on the EC_50_, 10 µM concentration was selected for the subsequent experiments. Such a cytotoxic effect was corroborated by the trypan blue exclusion assay (Figure 1C), excluding possible artifacts due to redox interference of TR with MTT reduction. Furthermore, 10 µM TR did not affect significantly the viability of PMBC, showing that TR exhibits selectivity against leukemia tumor cells in relation to normal human blood cells (Figure 1D). Additionally, morphological alterations were observed in Jurkat cells incubated with TR (Figure 1E–G). In flow cytometry, the frontal dispersion of laser (forward scatter, FSC) provides information about the relative cell size, and the lateral dispersion (side scatter, SSC) is related to cell granularity or complexity. By using the data from these parameters, it was shown that 10 µM TR incubated with Jurkat cells for 24 h decrease of cell size and increased granularity, defining a “live” and “dead” cell population (Figure 1E), whose quantification was presented in Figure 1F. Optical microscopy revealed that 10 µM TR promoted remarkable morphological changes in Jurkat cells characterized by loss of cell-cell adhesion, cell shrinkage, and plasma membrane disruption (Figure 1G).

### 3.2. TR Induced Caspase-Dependent Apoptosis in Jurkat Cells

**Figure 2:**
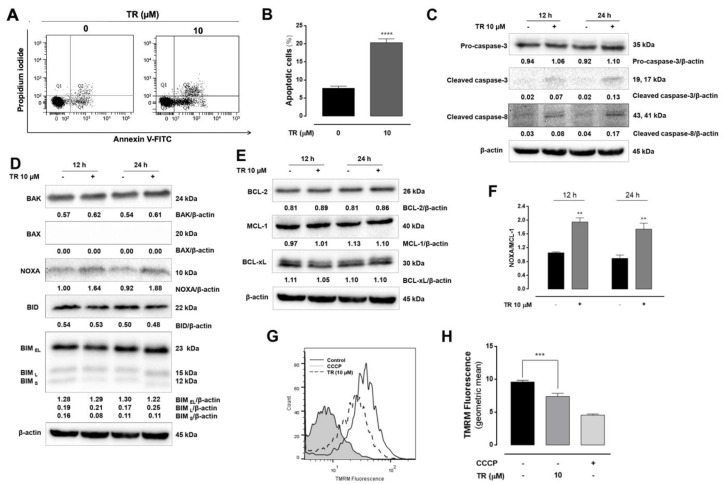
TR induces mitochondrial-dependent apoptosis in human leukemia Jurkat cells. Cell death profile was analyzed by double staining flow cytometric analysis with annexin V-FITC and propidium iodide in Jurkat cells after 24 h incubation with 10 µM TR. (**A**) Representative dot plot of at least three independent experiments performed in duplicate. (**B**) Quantification of apoptotic (annexin V-FITC positive) cells. The results are presented as the mean ± SEM of at least three independent experiments performed in duplicate. **** (*p* < 0.0001) indicates a difference from control (absence of the drug). Whole cell lysates from Jurkat cells treated with 10 µM TR for indicated time periods were subjected to Western blot analysis for (**C**) Expression levels of pro-caspase-3, cleaved-caspase-3 (Asp175) and cleaved caspase-8 (Asp391). (**D**) Expression levels of pro-apoptotic BCL-2 family proteins and (**E**) Expression levels of anti-apoptotic BCL-2 family proteins. Densitometry measurements, normalized to β-actin are indicated below the corresponding blot. The data are represented as the mean of at least three independent experiments. (**F**) Bar graphs shown intensity levels of ratio NOXA/MCL-1. The results are presented as the mean ± SEM of at least three independent experiments. ** (*p* < 0.01) indicates a difference from control (absence of the drug). (**G**) Representative histograms of TMRM fluorescence obtained by flow cytometry. Gray line (CCCP), dashed black line (10 µM TR) and black line (control). (**H**) Quantification of TMRM fluorescence. The results are presented as the mean ± SEM of at least three independent experiments. *** (*p* < 0.001) indicates a difference from control (absence of the drug).

To further characterize the cell death induced by TR in Jurkat cells, the annexin V-FITC/PI double staining flow cytometry was employed. The incubation of cells with 10 µM TR for 24 h resulted in increased annexin V-FITC stained Jurkat cells (annexin V^+^/PI^−^ and annexin V^+^/PI^+^), indicative of apoptosis (Figure 2A). The quantification of annexin V positive cells considering all replicates revealed a significant increase (*p* < 0.0001) of apoptotic cells induced by TR compared to control (Figure 2B). Moreover, TR increased the levels of active caspase-8 and caspase-3 (Figure 2C). The effects of TR on the expression of pro-apoptotic (Figure 2D) and anti-apoptotic (Figure 2E) proteins of Bcl-2 family showed an increased expression of BAK and NOXA. Considering Jurkat cells did not express BAX [20], we showed the TR disturbed the balance among pro- and anti-apoptotic proteins toward apoptosis induction as shown by increased NOXA/MCL-1 ratio (Figure 2F), although the expression of other BCL-2 family proteins remained unaltered. Alterations in expression and location of BCL-2 proteins are associated with mitochondrial permeabilization associated to the apoptotic process [21]. Thus, the effects of TR on the mitochondrial transmembrane potential (ΔΨ) were analyzed using TMRM, a cell-permeant dye that accumulates in active mitochondria with high membrane potential. TR dissipated the ΔΨ in Jurkat cells, as shown by the decreased the signal intensity of TMRM compared to the control (Figure 2G). The uncoupler CCCP was used to obtain the maximal dissipation of the ΔΨ and the quantification of replicates was presented in Figure 2H. These results indicated that TR induced caspase-dependent apoptosis and NOXA probably plays a regulatory role in cell death by inhibiting the anti-apoptotic activity of MCL-1 in leukemia Jurkat cells.

### 3.3. TR Stimulates Autophagy in Jurkat Cells

**Figure 3:**
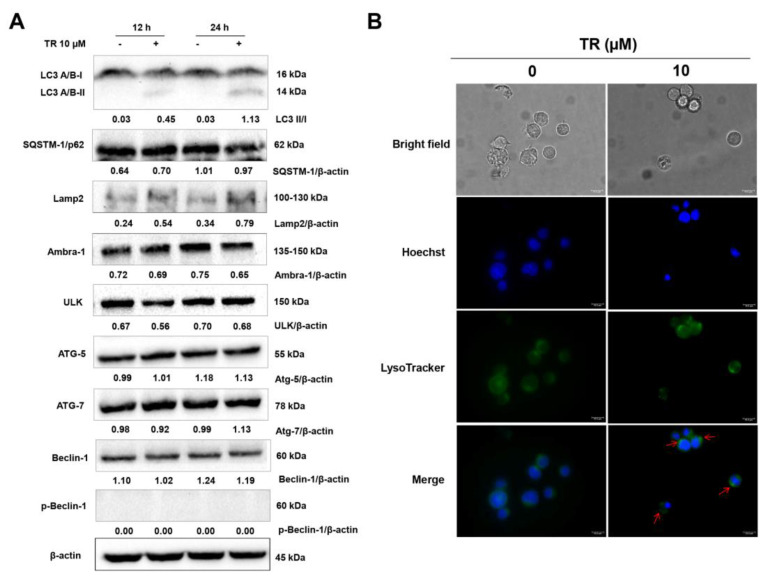
TR triggered autophagy in human leukemia Jurkat cells. (**A**) Whole cell lysates from Jurkat cells treated with 10 µM TR for indicated time periods were subjected to Western blot analysis for autophagy-related proteins expression levels. Densitometry measurements, normalized to β-actin are indicated below the corresponding blot. The data are represented as the mean of at least three independent experiments. (**B**) Jurkat cells were incubated with 10 µM TR for 24 h, followed by stained simultaneously with 50 nM LysoTracker Green and 5 µM Hoechst33342 in culture media for 30 min and analyzed by fluorescence microscopy (magnification 1000×, scale bars 10 µM).

Under cytotoxic stimuli, cells alter the autophagic flux as a protective response to promote cell survival [22,23]. However, an overstimulation of autophagy can result in cell death. In order to investigate whether modulation of autophagy was involved in the TR-induced cytotoxicity in Jurkat cells, we examined the expression of several autophagy-related proteins by Western blot after 12 and 24 h incubation with 10 μM TR. As shown in Figure 3A, TR induced the processing of full-length LC3-I to LC3-II in a time-dependent manner. Moreover, TR also increased the expression of LAMP2, a lysosomal membrane protein involved in lysosomal stability and autophagy. Since lysosomes fuse with the autophagosome during the autophagic process, we used the cell permeable fluorescent LysoTracker dye, which accumulates within the lysosomes at nanomolar concentrations. As shown in Figure 3B, the incubation of TR treatment enhanced fluorescence in Jurkat cells loaded with LysoTracker, with intracellular punctuated staining pattern, suggesting significant increase in lysosome numbers during the autophagic process.

### 3.4. Inhibition of Autophagy Enhanced TR-Induced Apoptosis in Human Leukemia Jurkat Cells

**Figure 4:**
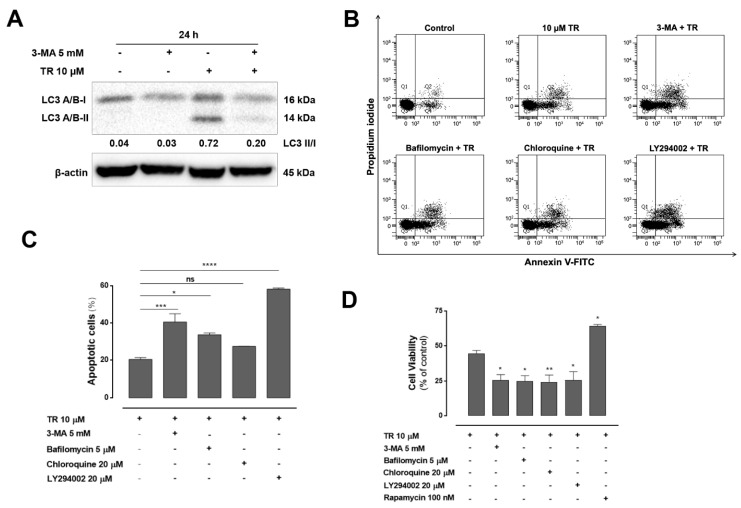
Inhibition of autophagy enhanced TR-Induced apoptosis in human leukemia Jurkat cells. (**A**) Whole cell lysates from Jurkat cells pretreated with 5 mM 3-MA for 1 h and incubated with 10 µM TR for another 24 h were subjected to Western blot analysis for LC3 A/B. Quantification results of LC3 II/I are indicated below the corresponding blot. The data are represented as the mean of at least three independent experiments. (**B**) Cell death profile was analyzed by double staining flow cytometric analysis with annexin V-FITC and propidium iodide in Jurkat cells after 24 h incubation with 10 µM TR in the absence or presence of 5 mM 3-MA, 5 µM bafilomycin A1, 20 µM chloroquine and 20 µM LY294002. Representative dot plot of at least three independent experiments performed in duplicate. (**C**) Quantification of apoptotic (annexin V-FITC positive) cells. The results are presented as the mean ± SEM of at least three independent experiments performed in duplicate. * (*p* < 0.05), *** (*p* < 0.001) and **** (*p* < 0.0001) indicates a difference from TR alone. (**D**) Cells were pretreated with 5 mM 3-MA, 5 µM bafilomycin A1, 20 µM chloroquine, 20 µM LY 294,002 and 100 nM rapamycin for 1 h and incubated with 10 µM TR for another 24 h. Cell viability assessed by the MTT reduction test. The results are presented as mean ± SEM of at least three independent experiments performed in triplicate. * (*p* < 0.05) and ** (*p* < 0.01) indicates a difference from TR alone.

Since autophagy has dual roles in regulating cell survival and cell death, we investigated the effect of TR-induced autophagy on apoptosis in Jurkat cells. Cells were pretreated for 1 h with 3-methyladenine (3-MA), an autophagy inhibitor that blocks the conversion of LC3-I to LC3-II and autophagosome formation in the presence or absence of TR for 24 h. Western blot analysis showed that TR treatment increased the protein levels of LC3-II, which were diminished by the addition of 3-MA (Figure 4A). Moreover, combined treatment of 3-MA and TR significantly increased cell population positive for annexin V-FITC compared to TR (Figure 4B,C). In addition, the autophagy inhibitors 3-MA, bafilomycin A1, chloroquine, and LY294002 significantly increased the cytotoxicity of TR in ALL cells, and the combination of the autophagy activator rapamycin and TR significantly attenuated it compared to TR (Figure 4D). These results suggested that inhibition of autophagy may sensitize cells to TR-induced apoptosis.

### 3.5. Modulation of Signaling Pathways Related to the Balance Between Proliferation and Death in Jurkat Cells by TR: PI3K/AKT/mTOR and Ras/Raf/MEK/ERK Cascades

**Figure 5:**
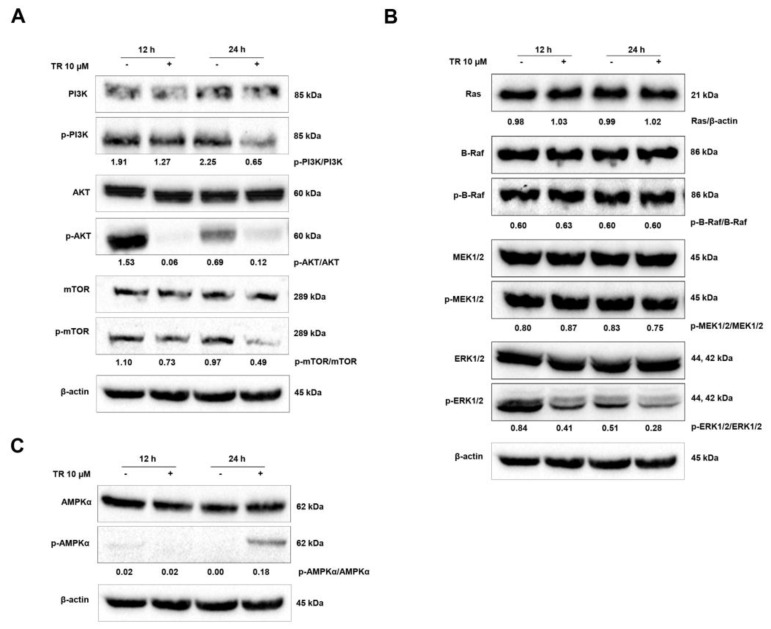
TR promotes apoptosis and autophagy through inhibition of PI3K/AKT/mTOR and Ras/Raf/MEK/ERK signaling and activation of AMPK in human leukemia Jurkat cells. Whole cell lysates from Jurkat cells treated with 10 µM TR for indicated time periods were subjected to Western blot analysis and investigated for (**A**) PI3K, p-PI3K (Tyr199/458), AKT, p-AKT (Ser473), mTOR, p-mTOR (Ser2228) proteins expression levels, (**B**) Ras, B-Raf, p-B-Raf (Ser445), MEK, p-MEK (Ser217/221), ERK, p-ERK (Thr202/Tyr204) proteins expression levels and (**C**) AMPK, p-AMPK (Thr172) proteins expression levels. Quantification results of p-PI3K/PI3K, p-AKT/AKT, p-mTOR/mTOR, Ras/β-actin, p-B-Raf/Raf, p-MEK/MEK and p-ERK/ERK are indicated below the corresponding blot. The data are represented as the mean of at least three independent experiments.

The PI3K/AKT/mTOR signaling pathway plays an important role in regulating cell cycle, proliferation, apoptosis, and autophagy [22]. To examine the involvement of the PI3K/AKT/mTOR pathway in TR-induced apoptosis and autophagy, we tested the activation of PI3K, AKT and mTOR by Western blot assay using phosphorylated antibodies. As shown in Figure 5A, treatment with TR decreased the levels of constitutively phosphorylated PI3K, AKT, and mTOR in Jurkat cells. It has been proposed by others that activation of ERK1/2 plays a pivotal role in cellular proliferation and in the control of cell cycle, and its inhibition is associated with apoptosis [24,25]. Thus, in order to evaluate the effects of TR on the MAPK/ERK signaling pathway, the expression of RAS, B-RAF, p-B-RAF, MEK, p-MEK, ERK, and p-ERK was analyzed by Western blot. Our data show that the MAPK/ERK pathway is constitutively active in ALL Jurkat cells and, interestingly, TR inhibits only the activation of ERK (p-ERK) at 12 and 24 h (Figure 5B). It remains to be elucidated whether TR interacts directly with the phosphorylation site of ERK or acts indirectly to promote such effect, since ERK signaling is suggested as an Achilles hell in cancer cells [26]. In parallel, we evaluated one of the central sensors/regulators of cellular metabolism in eukaryotes, the AMP-activated protein kinase (AMPK). It was shown that AMPK was activated by TR in Jurkat cells, as monitored by the phosphorylation of AMPK (Thr172) in 24 h (Figure 5C). These results show that AMPK activation and PI3K/AKT/mTOR suppression drive the autophagic response of ALL cells to TR exposure. Additionally, the inhibition of the downstream executioner of MAPK/ERK pathway (p-ERK) by TR might contribute to its antiproliferative/cytotoxic effects.

## 4. Discussion

**Figure 6:**
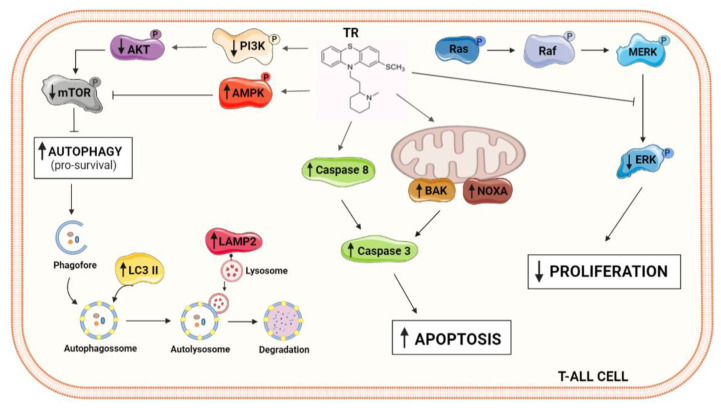
Schematic representative illustrations showing the molecular alterations promoted by TR in ALL Jurkat cells resulting in cell death.

The antipsychotic phenothiazine derivative thioridazine has exhibited potent antitumor activity in several tumor cells in vitro and cancer models in vivo [5,27]. Despite ongoing efforts to elucidate its mechanism of action, the underlying molecular mechanisms of TR-induced cell death still remain elusive. In this study, the role of autophagy in TR-induced apoptosis in human T-cell acute lymphoblastic leukemia model was exploited. Interestingly, the EC_50_ for Jurkat cells (10.7 µM) has no significant effect on the viability of normal cells. Such plasma concentration was easily achieved with a 50 mg TR oral dose in patients with acute myeloid leukemia (AML) patients in a recent clinical trial combining TR and cytarabine [28]. The activation of caspase-8 and the executioner caspase-3 indicated a caspase-dependent cell death. Furthermore, TR triggered autophagy and its inhibition enhanced the cytotoxic action of TR. TR-induced autophagy in Jurkat cells occurred through the suppression of PI3K/AKT/mTOR and Ras/Raf/MEK/ERK signaling pathways (Figure 6).

Tumor cells often develop resistance to the apoptotic stimuli, which is related to cancer progression. The balance between pro- and antiapoptotic proteins of Bcl-2 family is crucial to the regulation of apoptosis [29]. The NOXA/MCL-1 ratio seems to play a role in the cell fate upon chemotherapy [30]. The upregulation of NOXA induced by TR contributed to the triggering of apoptosis in Jurkat cells. In this regard, Horing et al. proposed that the pharmacological modulation of NOXA and MCL-1 is an effective for the treatment of mantle cell lymphoma [31]. It was shown that the NOXA/MCL-1 ratio has an important role in glucose limitation-mediated apoptosis [32] and also in the pancreatic cancer cell proliferation [33]. The NOXA/MCL-1 ratio is especially important in Jurkat, since these cells do not express BAX due to frameshift mutations resulting in premature termination of translation [20]. Then, BAX/BCL-2 ratio cannot be used as marker in this cell line [34]. Nevertheless, TR-induced apoptosis in Jurkat cells was mediated by the activation of the effector caspase-3.

The comprehension of the intricate relation between apoptosis and autophagy is essential for the development of effective cancer therapeutics [35]. Autophagy is a cellular process responsible for the renewal/elimination of organelles and proteins through the formation of autophagosomes and degradation by lysosomal hydrolases to maintain cell homeostasis [16]. Depending on the cell type and the stimulus, autophagy may act as a “double-edged sword”, acting on the regulation of cell survival/proliferation or inducing autophagic cell death [36]. In cancer cells, autophagy might be activated as a survival mechanism to resist chemotherapeutic agents induced apoptosis [37]. Due to this, several studies proposed that the inhibition of autophagy increased chemically-induced and consequently the antitumor activity of drugs [38,39,40]. Here we showed that TR induced an autophagic response in T-ALL cells. The expression of LC3-II, a marker of the autophagic flux required for elongation of autophagosomes [41], increased in a time-dependent manner after treatment with TR. In addition, the expression of LAMP2, a lysosomal membrane protein involved in final maturation and fusion of autophagosomes with lysosomes to form an autolysosome [42] was also increased. In an effort to determine whether TR-induced autophagy in Jurkat is acting a pro-survival process or it is associated with apoptosis, pharmacological tools (3-MA, chloroquine, bafilomycin A1 and LY294002) to inhibit autophagy were employed. The suppression of autophagy using all inhibitors significantly enhanced the TR-induced apoptosis in Jurkat cells. In accordance to this, previous studies showed that autophagy inhibition enhanced the antitumor effects of daunorubicin [43], dasatinib [44], and asparaginase [45] in leukemia models. Therefore, since autophagy stimulation is among the pro-survival strategies used by cancer cells to protect against cellular stress, it represents a promising therapeutic target.

To further advance mechanistical aspects of TR-induced cell death in T-ALL cells, signaling pathways related to apoptosis and autophagy were investigated. The PI3K/AKT/mTOR signaling pathway is crucial for the maintenance of cell homeostasis under normal physiological conditions [24]. However, it is frequently deregulated in numerous human cancers, including myeloid leukemia [46], hepatocellular carcinoma cells [47], and T-ALL [48]. Aberrant regulation of PI3K/AKT/mTOR pathway provides a proliferative advantage to tumor cells and it contributes to drug resistance development. Thus, the inhibition of the PI3K/AKT/mTOR signaling pathway might represent a possible therapeutic strategy for cancer treatment. TR decreased the levels of constitutively phosphorylated PI3K, AKT, and mTOR in Jurkat cells. This was also shown in ovarian cancer cells [8] and cervical and endometrial cancer cells [9]. Yet, recent studies showed that other structurally related antipsychotic phenothiazine derivatives also induced autophagy in tumor cells in vitro through the inhibition of PI3K/AKT/mTOR [49,50,51]. Interestingly, it was shown that normal T lymphocytes are not affected by PI3K/AKT/mTOR inhibitors [52]. The metabolic regulator AMPK was activated by TR. AMPK regulates multiple cellular processes, including autophagy, since active AMPK inhibits mTOR, a protein kinase that negatively regulates autophagy [53]. Although TR triggered a protective autophagic response in T-ALL cells, an antihistamine phenothiazine derivative promethazine induced autophagy-associated cell death in a Philadelphia chromosome-positive chronic myeloid leukemia model (K562) mediated by activation of AMPK [54], i.e., promethazine-induced autophagy in CML contributed to cell death. Thus, it is not clear yet whether these differences are due to the type of leukemia cell or the type and intensity of phenothiazine derivative promoting cell death. These open questions will be further investigated.

Another signaling pathway that regulates diverse cellular functions, including cell proliferation, survival, and differentiation, is the mitogen-activated protein kinase pathways (MAPK) [55,56,57]. Like PI3K/AKT/mTOR, Ras/Raf/MEK/ERK cascade is one of the major survival/proliferative signaling pathway that is often constitutively activated in cancer. Additionally, since there is a crosstalk between MAPK and PI3K signaling pathways, the simultaneous inhibition of these pathways may be an effective potential strategy for the treatment of cancer [58]. It is noteworthy that TR suppressed both pathways, which guarantee the cytotoxic efficiency of TR, despite the activation of a pro-survival autophagy T-ALL cells. The specific inhibition of ERK activation in MAPK-ERK pathway by TR deserves a further investigation, since ERK is considered an important target in cancer therapy [59].

In conclusion, our data show that TR inhibited cell proliferation and induced apoptosis and autophagy through inhibiting the PI3K/AKT/mTOR and Ras/Raf/MEK/ERK signaling pathway in acute lymphoblastic leukemia cells. Moreover, inhibition of autophagy enhanced the cytotoxicity of TR in Jurkat cells. The induction of autophagy may be an adaptive response to chemicals and antitumor drugs with a protective role in acute lymphoblastic leukemia, which might be considered as targetable for drug development purposes.

## Supplementary Materials

The following are available online at https://www.mdpi.com/article/10.3390/life11040365/s1, Figure S1: Western blots and densitometry analysis of pro-caspase-3, cleaved-c aspase-3, cleaved-caspase-8, pro- and anti-apoptotic BCL-2 family proteins in Jurkat cells, Figure S2: Western blots and densitometry analysis of autophagy-related proteins in Jurkat cells, Figure S3: Western blots and densitometry analysis of LC3 A/B protein in Jurkat cells, Figure S4: Western blots and densitometry analysis of PI3K/AKT/mTOR, Ras/Raf/MEK/ERK and AMPK signaling pathway in Jurkat cells.

## References

[1] Pui C.H., Robison L.L., Look A.T. (2008). Acute lymphoblastic leukaemia. Lancet.

[2] Ferrando A.A., Neuberg D.S., Staunton J., Loh M.L., Huard C., Raimondi S.C., Behm F.G., Pui C.H., Downing J.R., Gilliland D.G. (2002). Gene expression signatures define novel oncogenic pathways in T cell acute lymphoblastic leukemia. Cancer Cell.

[3] Lehmann H.E., Ban T.A. (1997). The history of the psychopharmacology of schizophrenia. Can. J. Psychiatry.

[4] Min K.J., Seo B.R., Bae Y.C., Yoo Y.H., Kwon T.K. (2014). Antipsychotic agent thioridazine sensitizes renal carcinoma Caki cells to TRAIL-induced apoptosis through reactive oxygen species-mediated inhibition of akt signaling and downregulation of Mcl-1 and c-FLIP(L). Cell Death Dis..

[5] Varga B., Csonka A., Csonka A., Molnar J., Amaral L., Spengler G. (2017). Possible biological and clinical applications of phenothiazines. Anticancer Res..

[6] de Faria P.A., Bettanin F., Cunha R.L., Paredes-Gamero E.J., Homem-de-Mello P., Nantes I.L., Rodrigues T. (2015). Cytotoxicity of phenothiazine derivatives associated with mitochondrial dysfunction: A structure-activity investigation. Toxicology.

[7] Michał Otręba M., Kośmider L. (2021). In vitro anticancer activity of fluphenazine, perphenazine and prochlorperazine. A review. J. Appl. Toxicol..

[8] Rho S.B., Kim B.R., Kang S. (2011). A gene signature-based approach identifies thioridazine as an inhibitor of phosphatidylinositol-3’-kinase (PI3K)/AKT pathway in ovarian cancer cells. Gynecol. Oncol..

[9] Kang S., Dong S.M., Kim B.R., Park M.S., Trink B., Byun H.J., Rho S.B. (2012). Thioridazine induces apoptosis by targeting the PI3K/Akt/mTOR pathway in cervical and endometrial cancer cells. Apoptosis.

[10] Zhelev Z., Ohba H., Bakalova R., Hadjimitova V., Ishikawa M., Shinohara Y., Baba Y. (2004). Phenothiazines suppress proliferation and induce apoptosis in cultured leukemic cells without any influence on the viability of normal lymphocytes. Phenothiazines and leukemia. Cancer Chemother. Pharm..

[11] Rai S., Tanaka H., Suzuki M., Espinoza J.L., Kumode T., Tanimura A., Yokota T., Oritani K., Watanabe T., Kanakura Y. (2020). Chlorpromazine eliminates acute myeloid leukemia cells by perturbing subcellular localization of FLT3-ITD and KIT-D816V. Nat. Commun..

[12] Claudia Tregnago C., Da Ros A., Porcù E., Benetton M., Simonato M., Simula L., Borella G., Polato K., Minuzzo S., Borile S. (2020). Thioridazine requires calcium influx to induce MLL-AF6-rearranged AML cell death. Blood Adv..

[13] Buckley N.A., Whyte I.M., Dawson A.H. (1995). Cardiotoxicity more common in thioridazine overdose than with other neuroleptics. J. Toxicol. Clin. Toxicol..

[14] Zhou J., Yang J., Fan X., Hu S., Zhou F., Dong J., Zhang S., Shang Y., Jiang X., Guo H. (2016). Chaperone-mediated autophagy regulates proliferation by targeting RND3 in gastric cancer. Autophagy.

[15] Xia T., Wang J., Wang Y., Wang Y., Cai J., Wang M., Chen Q., Song J., Yu Z., Huang W. (2016). Inhibition of autophagy potentiates anticancer property of 20(S)-ginsenoside Rh2 by promoting mitochondria-dependent apoptosis in human acute lymphoblastic leukaemia cells. Oncotarget.

[16] Yang Z., Klionsky D.J. (2010). Eaten alive: A history of macroautophagy. Nat. Cell Biol..

[17] Degenhardt K., Mathew R., Beaudoin B., Bray K., Anderson D., Chen G., Mukherjee C., Shi Y., Gelinas C., Fan Y. (2006). Autophagy promotes tumor cell survival and restricts necrosis, inflammation, and tumorigenesis. Cancer Cell.

[18] Rubinsztein D.C., Codogno P., Levine B. (2012). Autophagy modulation as a potential therapeutic target for diverse diseases. Nat. Rev. Drug Discov..

[19] Yang Z.J., Chee C.E., Huang S., Sinicrope F.A. (2011). The role of autophagy in cancer: Therapeutic implications. Mol. Cancer Ther..

[20] Brimmell M., Mendiola R., Mangion J., Packham G. (1998). BAX frameshift mutations in cell lines derived from human haemopoietic malignancies are associated with resistance to apoptosis and microsatellite instability. Oncogene.

[21] Kalkavan H., Green D.R. (2018). MOMP, cell suicide as a BCL-2 family business. Cell Death Differ..

[22] Klionsky D.J., Abdelmohsen K., Abe A., Abedin M.J., Abeliovich H., Acevedo Arozena A., Adachi H., Adams C.M., Adams P.D., Adeli K. (2016). Guidelines for the use and interpretation of assays for monitoring autophagy (3rd edition). Autophagy.

[23] Amaravadi R.K., Thompson C.B. (2007). The roles of therapy-induced autophagy and necrosis in cancer treatment. Clin. Cancer Res..

[24] Franke T.F., Hornik C.P., Segev L., Shostak G.A., Sugimoto C. (2003). PI3K/Akt and apoptosis: Size matters. Oncogene.

[25] Meloche S., Pouyssegur J. (2007). The ERK1/2 mitogen-activated protein kinase pathway as a master regulator of the G1- to S-phase transition. Oncogene.

[26] Mebratu Y., Tesfaigzi Y. (2009). How ERK1/2 activation controls cell proliferation and cell death: Is subcellular localization the answer?. Cell Cycle.

[27] Spengler G., Csonka A., Molnar J., Amaral L. (2016). The anticancer activity of the old neuroleptic phenothiazine-type drug thioridazine. Anticancer Res..

[28] Aslostovar L., Boyd A.L., Almakadi M., Collins T.J., Leong D.P., Tirona R.G., Kim R.B., Julian J.A., Xenocostas A., Leber B. (2018). A phase 1 trial evaluating thioridazine in combination with cytarabine in patients with acute myeloid leukemia. Blood Adv..

[29] Czabotar P.E., Lessene G., Strasser A., Adams J.M. (2014). Control of apoptosis by the BCL-2 protein family: Implications for physiology and therapy. Nat. Rev. Mol. Cell Biol..

[30] Ploner C., Kofler R., Villunger A. (2008). Noxa: At the tip of the balance between life and death. Oncogene.

[31] Horing E., Montraveta A., Heine S., Kleih M., Schaaf L., Vohringer M.C., Esteve-Arenys A., Roue G., Colomer D., Campo E. (2017). Dual targeting of MCL1 and NOXA as effective strategy for treatment of mantle cell lymphoma. Br. J. Haematol..

[32] Alves N.L., Derks I.A., Berk E., Spijker R., van Lier R.A., Eldering E. (2006). The Noxa/Mcl-1 axis regulates susceptibility to apoptosis under glucose limitation in dividing t cells. Immunity.

[33] Naumann K., Schmich K., Jaeger C., Kratz F., Merfort I. (2012). Noxa/Mcl-1 balance influences the effect of the proteasome inhibitor MG-132 in combination with anticancer agents in pancreatic cancer cell lines. Anticancer Drugs.

[34] Perlman H., Zhang X., Chen M.W., Walsh K., Buttyan R. (1999). An elevated bax/bcl-2 ratio corresponds with the onset of prostate epithelial cell apoptosis. Cell Death Differ..

[35] Radogna F., Dicato M., Diederich M. (2015). Cancer-type-specific crosstalk between autophagy, necroptosis and apoptosis as a pharmacological target. Biochem. Pharm..

[36] Cheong H., Lu C., Lindsten T., Thompson C.B. (2012). Therapeutic targets in cancer cell metabolism and autophagy. Nat. Biotechnol..

[37] Janku F., McConkey D.J., Hong D.S., Kurzrock R. (2011). Autophagy as a target for anticancer therapy. Nat. Rev. Clin. Oncol..

[38] Zhu S., Cao L., Yu Y., Yang L., Yang M., Liu K., Huang J., Kang R., Livesey K.M., Tang D. (2013). Inhibiting autophagy potentiates the anticancer activity of ifn1@/ifnalpha in chronic myeloid leukemia cells. Autophagy.

[39] Liu S., Lu W., Li S., Li S., Liu J., Xing Y., Zhang S., Zhou J.Z., Xing H., Xu Y. (2017). Identification of JL1037 as a novel, specific, reversible lysine-specific demethylase 1 inhibitor that induce apoptosis and autophagy of AML cells. Oncotarget.

[40] Li M.L., Xu Y.Z., Lu W.J., Li Y.H., Tan S.S., Lin H.J., Wu T.M., Li Y., Wang S.Y., Zhao Y.L. (2018). Chloroquine potentiates the anticancer effect of sunitinib on renal cell carcinoma by inhibiting autophagy and inducing apoptosis. Oncol. Lett..

[41] Ozpolat B., Benbrook D.M. (2015). Targeting autophagy in cancer management-strategies and developments. Cancer Manag. Res..

[42] Saftig P., Klumperman J. (2009). Lysosome biogenesis and lysosomal membrane proteins: Trafficking meets function. Nat. Rev. Mol. Cell Biol..

[43] Han W., Sun J., Feng L., Wang K., Li D., Pan Q., Chen Y., Jin W., Wang X., Pan H. (2011). Autophagy inhibition enhances daunorubicin-induced apoptosis in K562 cells. PLoS ONE.

[44] Xie N., Zhong L., Liu L., Fang Y., Qi X., Cao J., Yang B., He Q., Ying M. (2014). Autophagy contributes to dasatinib-induced myeloid differentiation of human acute myeloid leukemia cells. Biochem. Pharm..

[45] Takahashi H., Inoue J., Sakaguchi K., Takagi M., Mizutani S., Inazawa J. (2017). Autophagy is required for cell survival under L-asparaginase-induced metabolic stress in acute lymphoblastic leukemia cells. Oncogene.

[46] Tamburini J., Elie C., Bardet V., Chapuis N., Park S., Broet P., Cornillet-Lefebvre P., Lioure B., Ugo V., Blanchet O. (2007). Constitutive phosphoinositide 3-kinase/Akt activation represents a favorable prognostic factor in de novo acute myelogenous leukemia patients. Blood.

[47] Yang J., Pi C., Wang G. (2018). Inhibition of PI3K/Akt/mTOR pathway by apigenin induces apoptosis and autophagy in hepatocellular carcinoma cells. Biomed. Pharm..

[48] Silva A., Yunes J.A., Cardoso B.A., Martins L.R., Jotta P.Y., Abecasis M., Nowill A.E., Leslie N.R., Cardoso A.A., Barata J.T. (2008). PTEN posttranslational inactivation and hyperactivation of the PI3K/Akt pathway sustain primary T cell leukemia viability. J. Clin. Investig..

[49] Shin S.Y., Lee K.S., Choi Y.K., Lim H.J., Lee H.G., Lim Y., Lee Y.H. (2013). The antipsychotic agent chlorpromazine induces autophagic cell death by inhibiting the Akt/mTOR pathway in human U-87MG glioma cells. Carcinogenesis.

[50] Wu C.H., Bai L.Y., Tsai M.H., Chu P.C., Chiu C.F., Chen M.Y., Chiu S.J., Chiang J.H., Weng J.R. (2016). Pharmacological exploitation of the phenothiazine antipsychotics to develop novel antitumor agents-A drug repurposing strategy. Sci. Rep..

[51] Jhou A.J., Chang H.C., Hung C.C., Lin H.C., Lee Y.C., Liu W.T., Han K.F., Lai Y.W., Lin M.Y., Lee C.H. (2021). Chlorpromazine, an antipsychotic agent, induces G2/M phase arrest and apoptosis via regulation of the PI3K/AKT/mTOR-mediated autophagy pathways in human oral cancer. Biochem. Pharm..

[52] Alameen A.A., Simioni C., Martelli A.M., Zauli G., Ultimo S., McCubrey J.A., Gonelli A., Marisi G., Ulivi P., Capitani S. (2016). Healthy CD4+ T lymphocytes are not affected by targeted therapies against the PI3K/Akt/mTOR pathway in T-cell acute lymphoblastic leukemia. Oncotarget.

[53] Herzig S., Shaw R.J. (2018). AMPK: Guardian of metabolism and mitochondrial homeostasis. Nat. Rev. Mol. Cell Biol..

[54] Medeiros H.C.D., Colturato-Kido C., Ferraz L.S., Costa C.A., Moraes V.W.R., Paredes-Gamero E.J., Tersariol I.L.S., Rodrigues T. (2020). AMPK activation induced by promethazine increases NOXA expression and Beclin-1 phosphorylation and drives autophagy-associated apoptosis in chronic myeloid leukemia. Chem. Biol. Interact..

[55] Roberts P.J., Der C.J. (2007). Targeting the RAF-MEK-ERK mitogen-activated protein kinase cascade for the treatment of cancer. Oncogene.

[56] Ward A.F., Braun B.S., Shannon K.M. (2012). Targeting oncogenic Ras signaling in hematologic malignancies. Blood.

[57] Chung E., Kondo M. (2011). Role of Ras/Raf/MEK/ERK signaling in physiological hematopoiesis and leukemia development. Immunol. Res..

[58] Kohno M., Pouyssegur J. (2006). Targeting the ERK signaling pathway in cancer therapy. Ann. Med..

[59] Feifei L., Xiaotong Y., Meiyu G., Min H. (2018). Targeting ERK, an Achilles’ Heel of the MAPK pathway, in cancer therapy. Acta Pharm. Sin. B.

