# Supplementary material for: Inhibition of Autophagy Enhances the Antitumor Effect of Thioridazine in Acute Lymphoblastic Leukemia Cells
Source: Life (Basel). 2021 Apr 20;11(4):365. doi: 10.3390/life11040365 (PMC8073363; doi:10.3390/life11040365)
Supplement: Supplementary file 1 [file life-11-00365-s001.zip › life-1142324-supplementary.pdf]

life
MDPI

Supplementary Materials: Inhibition of Autophagy Enhances the Antitumor Effect of Thioridazine in Acute Lymphoblastic Leukemia Cells

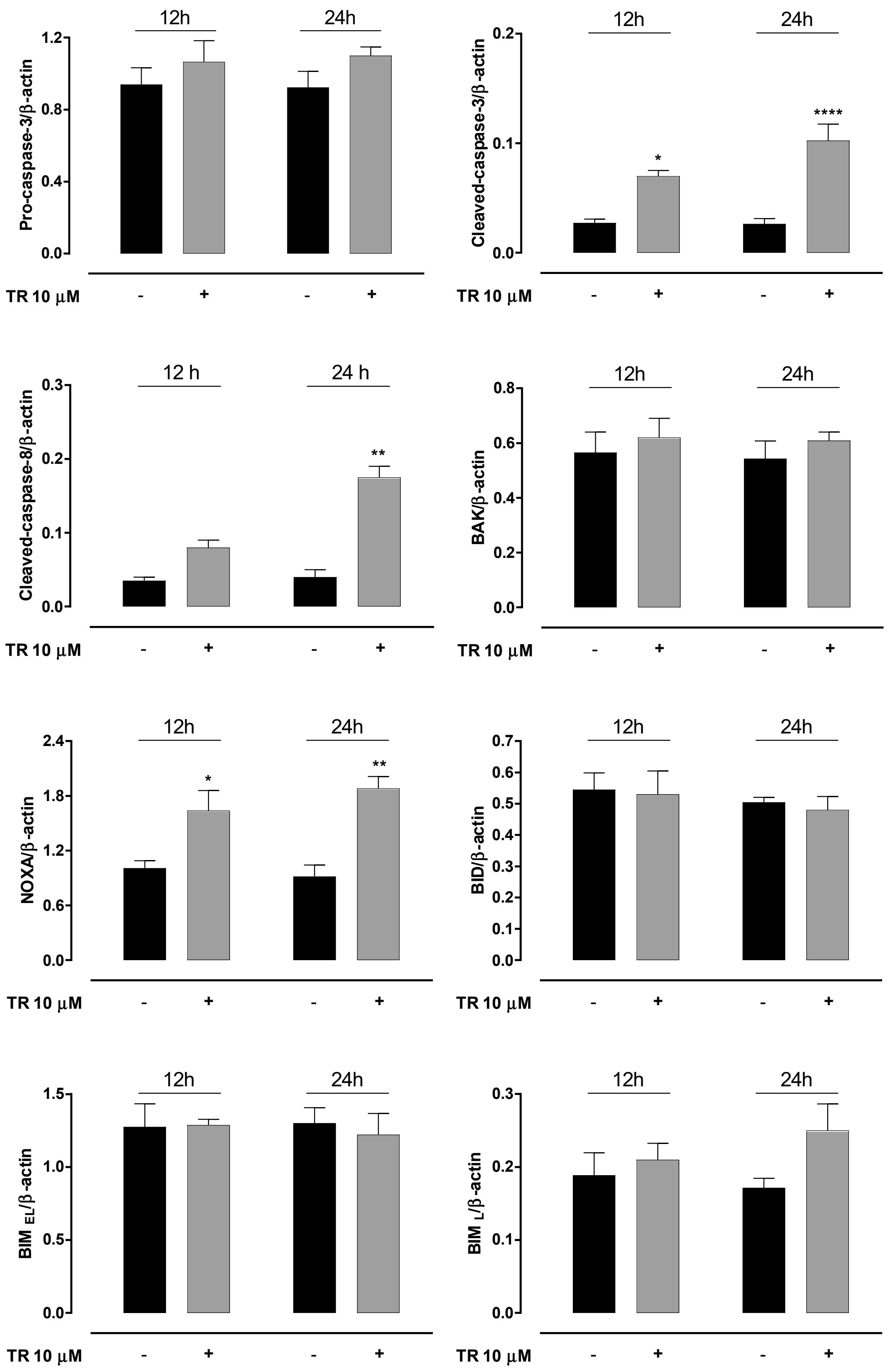

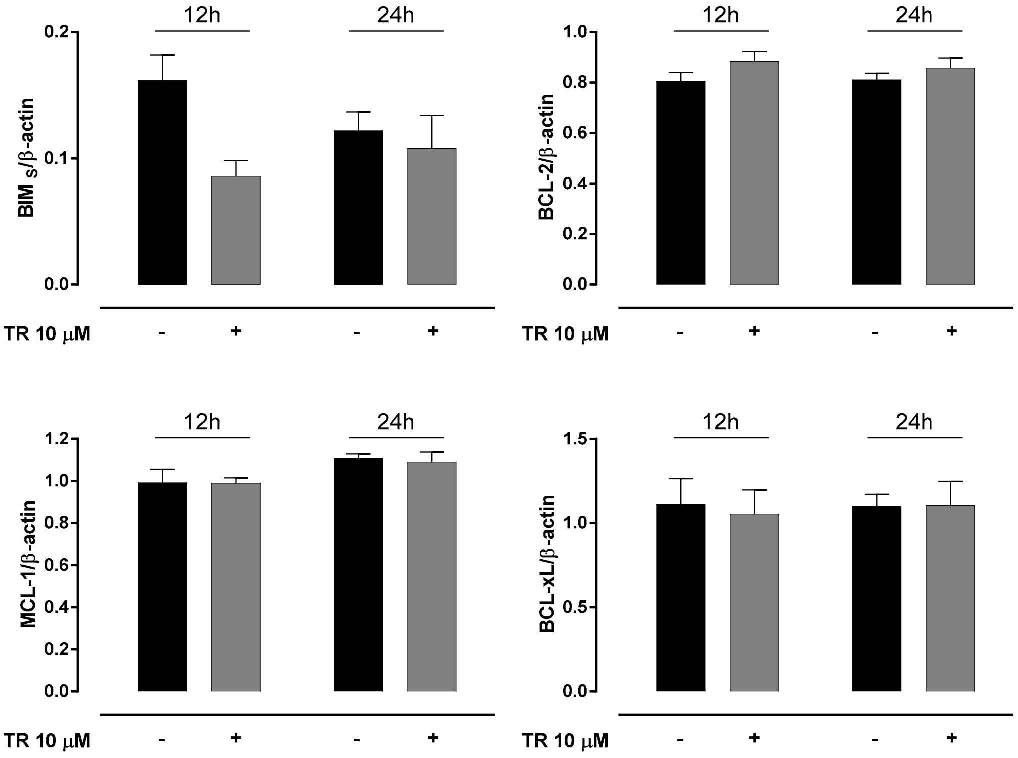

	Untreated 12 h	TR 10 μM 12 h	Untreated 24 h	TR 10 μM 24 h
**Pro-caspase-3/β-actin**	0.94	1.06	0.92	1.10
**Cleaved-caspase-3/β-actin**	0.02	0.07	0.02	0.13
**Cleaved-caspase-8/β-actin**	0.03	0.08	0.04	0.17
**BAK/β-actin**	0.57	0.62	0.54	0.61
**BAX/β-actin**	0.00	0.00	0.00	0.00
**NOXA/β-actin**	1.00	1.64	0.92	1.88
**BID/β-actin**	0.54	0.53	0.50	0.48
**BIM_{EL}/β-actin**	1.28	1.29	1.30	1.22
**BIM_{L}/β-actin**	0.19	0.21	0.17	0.25
**BIM_{S}/β-actin**	0.16	0.08	0.11	0.11
**BCL-2/β-actin**	0.81	0.89	0.81	0.86
**MCL-1/β-actin**	0.97	1.01	1.13	1.10
**BCL-xL/β-actin**	1.11	1.05	1.10	1.10

(**a**) Densitometry Readings/intensity Ratio.

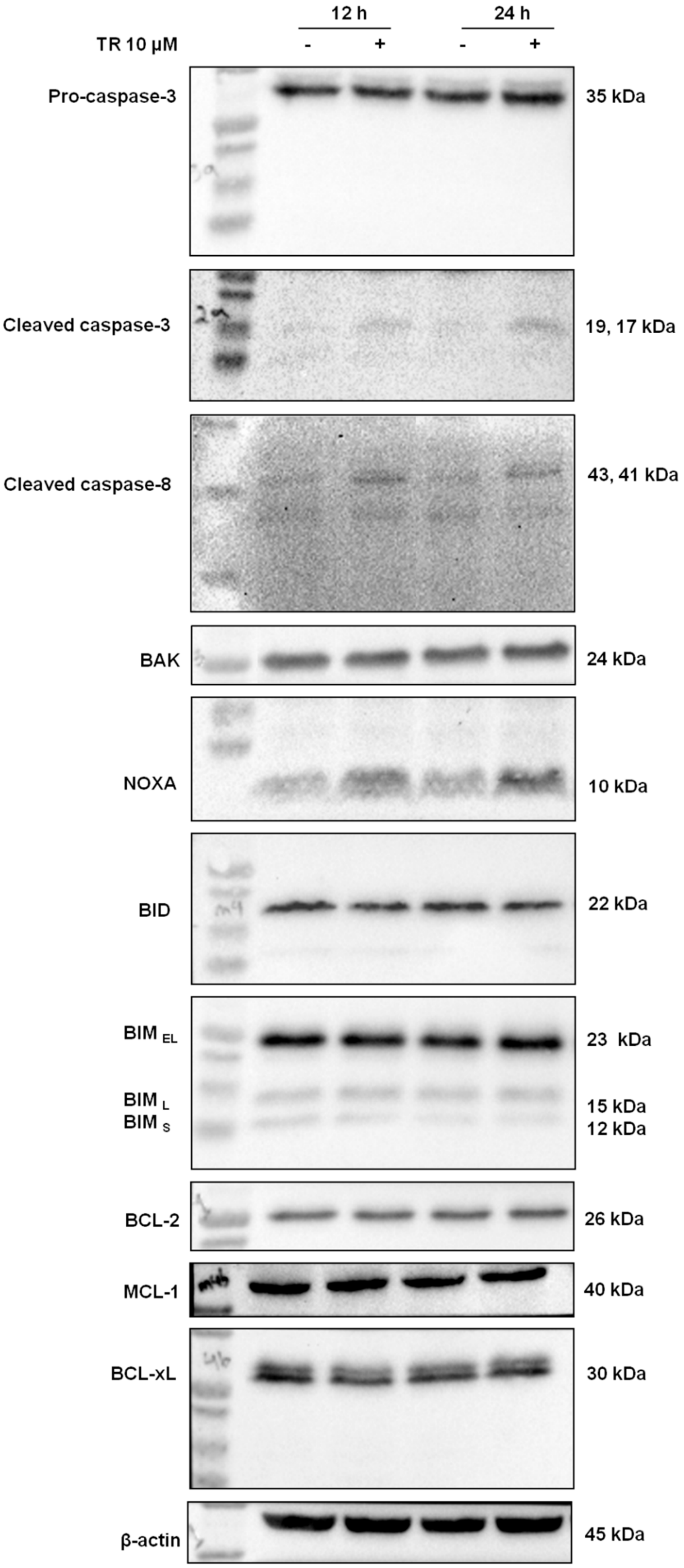

12 h
24 h
TR 10 μM
-
+
-
+
Pro-caspase-3
35 kDa
Cleaved caspase-3
19, 17 kDa
Cleaved caspase-8
43, 41 kDa
BAK
24 kDa
NOXA
10 kDa
BID
22 kDa
BIM EL
23 kDa
BIM L
15 kDa
BIM S
12 kDa
BCL-2
26 kDa
MCL-1
40 kDa
BCL-xL
30 kDa
β-actin
45 kDa

(b)

Figure S1. Western blots and densitometry analysis of pro-caspase-3, cleaved-caspase-3, cleaved-caspase-8, pro- and anti-apoptotic BCL-2 family proteins in Jurkat cells. Jurkat cells were treated with 10 μM TR for 12 and 24 h. An equal amount of protein was fractionated in 12 % polyacrylamide gels and transferred to nitrocellulose membranes. Band intensities of pro-caspase-3, cleaved-caspase-3 (Asp175), cleaved-caspase-8 (Asp391), pro-apoptotic (BAK, BAX, NOXA, BID, BIM), and anti-apoptotic (BCL-2, MCL-1, BCL-xL) proteins were analyzed by (**a**) densitometry readings/intensity ratio, using Image Lab™ software, version 5.0 (Bio-Rad Laboratories, Inc. USA), and were normalized to the corresponding β-actin value and (**b**) immunoblots, respectively.

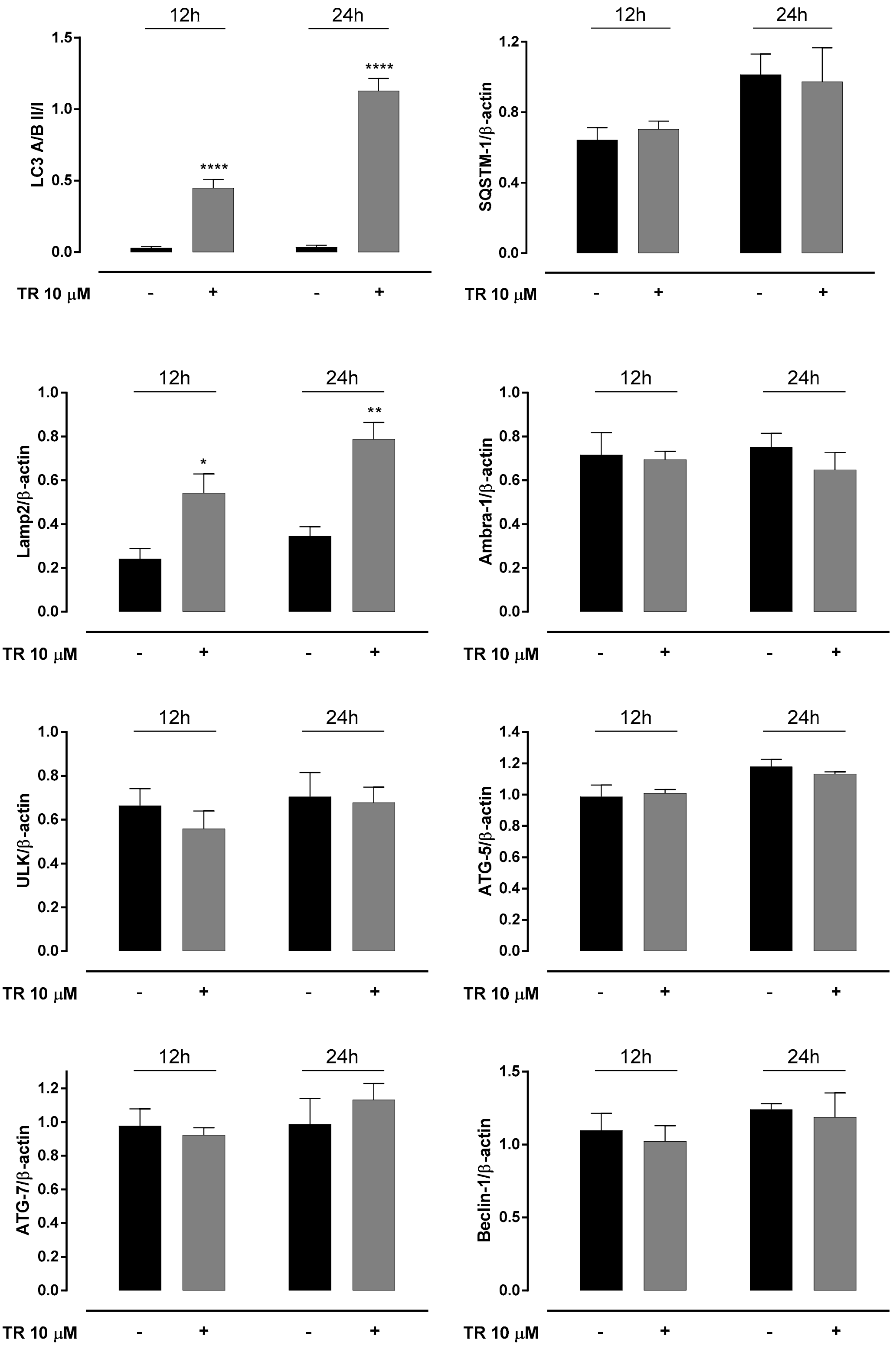

	**Untreated 12 h**	**TR 10 μM 12 h**	**Untreated 24 h**	**TR 10 μM 24 h**
**LC3 II/I**	0.03	0.45	0.03	1.13
**SQSTM-1/β-actin**	0.64	0.70	1.01	0.97
**Lamp-2/β-actin**	0.24	0.54	0.34	0.79
**Ambra-1/β-actin**	0.72	0.69	0.75	0.65
**ULK/β-actin**	0.67	0.56	0.70	0.68
**Atg-5/β-actin**	0.99	1.01	1.18	1.13
**Atg-7/β-actin**	0.98	0.92	0.99	1.13
**Beclin-1/β-actin**	1.10	1.02	1.24	1.19
**p-Beclin-1/β-actin**	0.00	0.00	0.00	0.00

(**a**) Densitometry Readings/intensity Ratio.

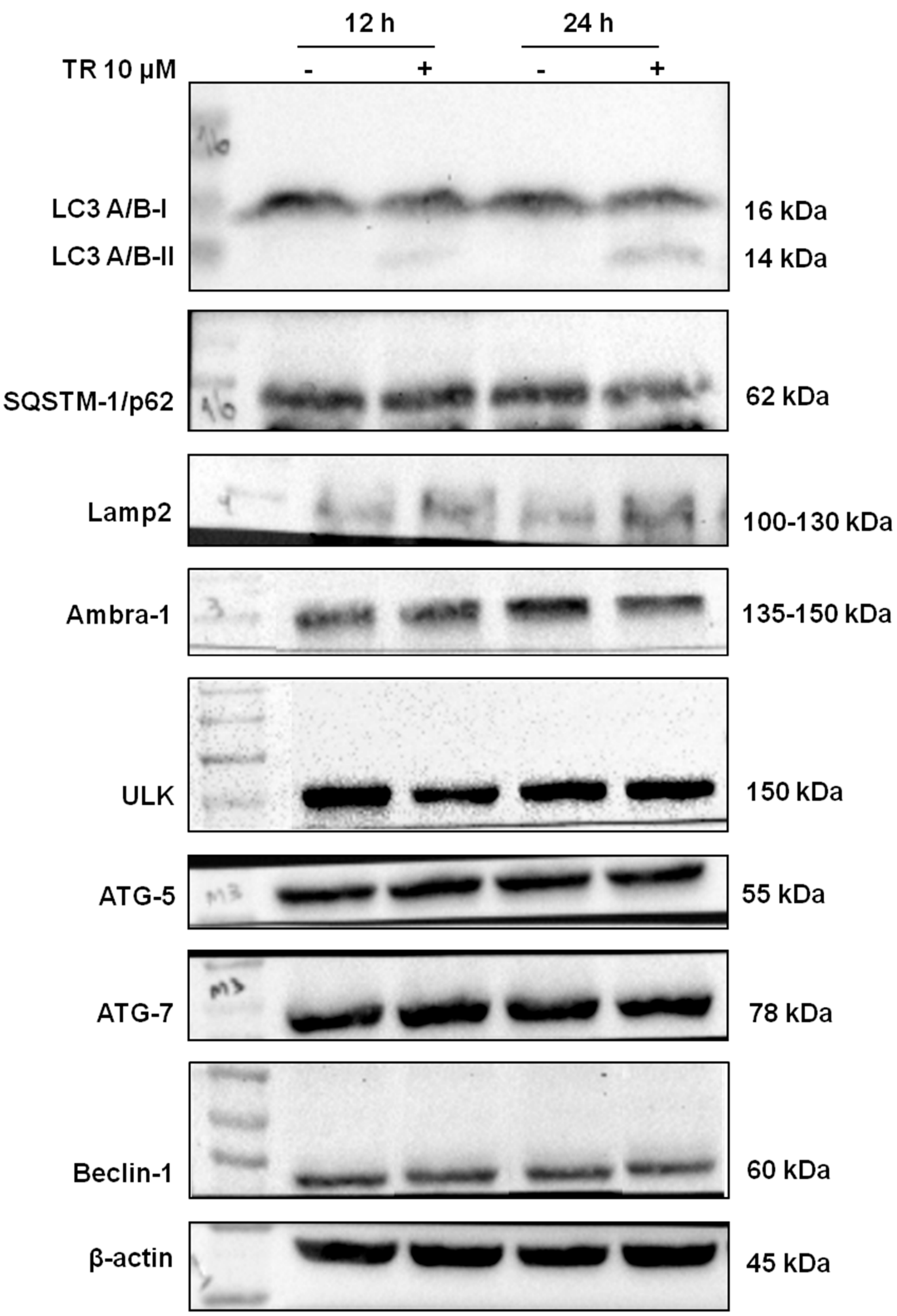

(**b**)

Figure S2. Western blots and densitometry analysis of autophagy-related proteins in Jurkat cells. Jurkat cells were treated with 10 μM TR for 12 and 24 h. An equal amount of protein was fractionated in 12 % polyacrylamide gels and transferred to nitrocellulose membranes. Band intensities of LC3, SQSTM-1, Lamp-2, Ambra-1, ULK, Atg-5, Atg-7, Beclin-1 and p-Beclin-1 proteins were analyzed by (**a**) densitometry readings/intensity ratio, using Image Lab™ software, version 5.0 (Bio-Rad Laboratories, Inc. USA), and were normalized to the corresponding β-actin value and (**b**) immunoblots, respectively.

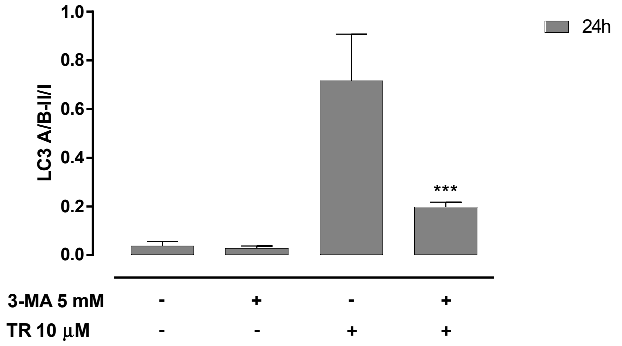

	Untreated	3-MA 5 mM	TR 10 μM	3-MA 5 mM + TR 10 μM
**LC3 II/I**	0.04	0.03	0.72	0.20

(**a**) Densitometry Readings/intensity Ratio

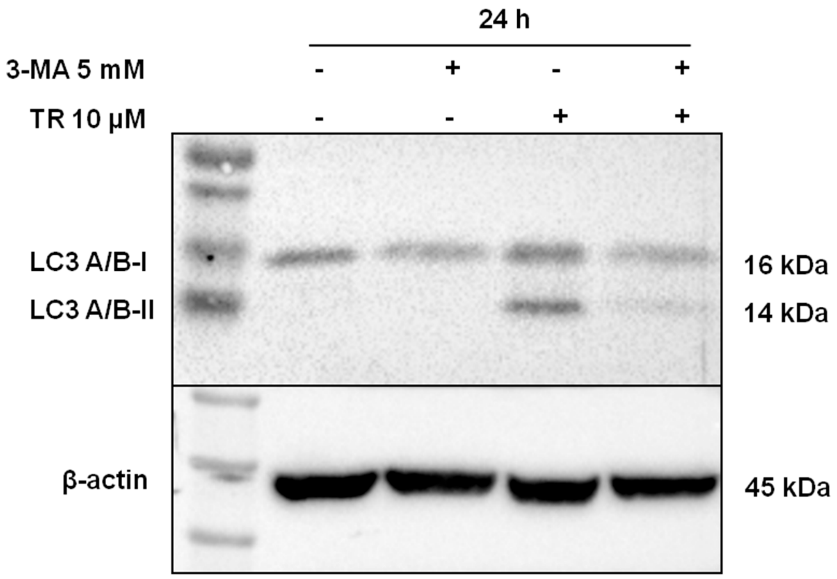

(**b**)

Figure S3. Western blots and densitometry analysis of LC3 A/B protein in Jurkat cells. Jurkat cells were pretreated with 5 mM 3-MA for 1 hour and incubated with 10 μM TR for another 24 h. An equal amount of protein was fractionated in 12 % polyacrylamide gels and transferred to nitrocellulose membranes. Band intensity of LC3 A/B protein was analyzed by (**a**) densitometry readings/intensity ratio, using Image Lab™ software, version 5.0 (Bio-Rad Laboratories, Inc. USA), and was normalized to the corresponding β-actin value and (**b**) immunoblots, respectively.

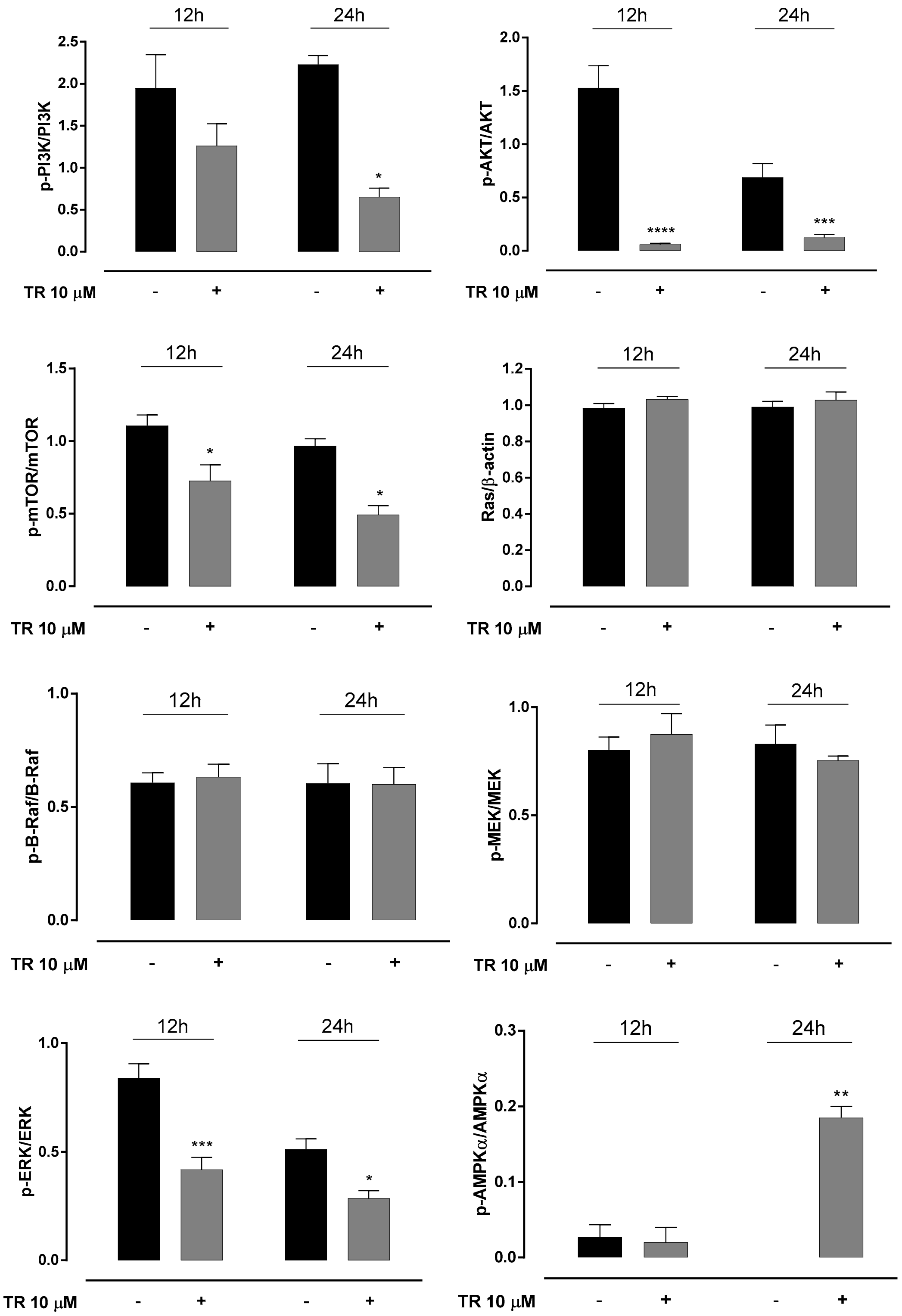
12h
24h
p-PI3K/PI3K
2.5
2.0
1.5
1.0
0.5
0.0
*
TR 10 μM
-
+
-
+
12h
24h
p-AKT/AKT
2.0
1.5
1.0
0.5
0.0
****
***
TR 10 μM
-
+
-
+
12h
24h
p-mTOR/mTOR
1.5
1.0
0.5
0.0
*
*
TR 10 μM
-
+
-
+
12h
24h
Ras/β-actin
1.2
1.0
0.8
0.6
0.4
0.2
0.0
TR 10 μM
-
+
-
+
12h
24h
p-B-Raf/B-Raf
1.0
0.5
0.0
TR 10 μM
-
+
-
+
12h
24h
p-MEK/MEK
1.0
0.5
0.0
TR 10 μM
-
+
-
+
12h
24h
p-ERK/ERK
1.0
0.5
0.0
***
*
TR 10 μM
-
+
-
+
12h
24h
p-AMPKα/AMPKα
0.3
0.2
0.1
0.0
**
TR 10 μM
-
+
-
+

	Untreated 12 h	TR 10 μM 12 h	Untreated 24 h	TR 10 μM 24 h
**p-PI3K/PI3K**	1.91	1.27	2.25	0.65
**p-AKT/AKT**	1.53	0.06	0.69	0.12
**p-mTOR/mTOR**	1.10	0.73	0.97	0.49
**Ras/β-actin**	0.98	1.03	0.99	1.02
**p-B-Raf/B-Raf**	0.60	0.63	0.60	0.60
**p-MEK1/2/MEK1/2**	0.80	0.87	0.83	0.75
**p-ERK1/2/ERK1/2**	0.84	0.41	0.51	0.28
**p-AMPKα/AMPKα**	0.02	0.02	0.00	0.18

(**a**) Densitometry Readings/intensity Ratio

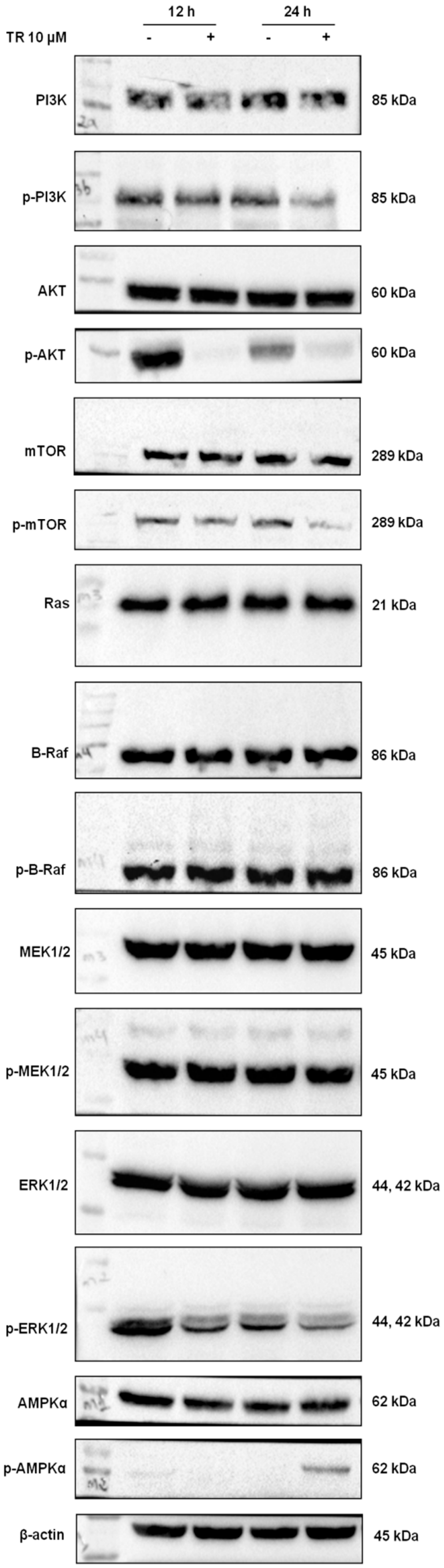

12 h
24 h
TR 10 μM
-
+
-
+
PI3K
85 kDa
p-PI3K
85 kDa
AKT
60 kDa
p-AKT
60 kDa
mTOR
289 kDa
p-mTOR
289 kDa
Ras
21 kDa
B-Raf
86 kDa
p-B-Raf
86 kDa
MEK1/2
45 kDa
p-MEK1/2
45 kDa
ERK1/2
44, 42 kDa
p-ERK1/2
44, 42 kDa
AMPKα
62 kDa
p-AMPKα
62 kDa
β-actin
45 kDa

(**b**)

Figure S4. Western blots and densitometry analysis of PI3K/AKT/mTOR, Ras/Raf/MEK/ERK and AMPK signaling pathway in Jurkat cells. Jurkat cells were treated with 10 μM TR for 12 and 24 h. An equal amount of protein was fractionated in 12 % polyacrylamide gels and transferred to nitrocellulose membranes. Band intensities of PI3K, p-PI3K (Tyr199/458), AKT, p-AKT (Ser473), mTOR, p-mTOR (Ser2228), Ras, B-Raf, p-B-Raf (Ser445), MEK, p-MEK (Ser217/221), ERK, p-ERK (Thr202/Tyr204), AMPKα and p-AMPKα (Thr172) proteins were analyzed by (**a**) densitometry readings/intensity ratio, using Image Lab™ software, version 5.0 (Bio-Rad Laboratories, Inc. USA), and were normalized to the corresponding β-actin value and (**b**) immunoblots, respectively.
